# To: Biomarkers of neuropsychiatric dysfunction in intensive care unit survivors: a prospective cohort study

**DOI:** 10.62675/2965-2774.20240260-en

**Published:** 2024-06-05

**Authors:** Josef Finsterer, Fulvio Alexandre Scorza

**Affiliations:** 1 Neurology and Neurophysiology Center Vienna Austria Neurology and Neurophysiology Center - Vienna, Austria.; 2 Universidade Federal de São Paulo Escola Paulista de Medicina Disciplina de Neurociência São Paulo SP Brazil Disciplina de Neurociência, Escola Paulista de Medicina, Universidade Federal de São Paulo - São Paulo (SP), Brazil.

**To the Editor**

We read with interest the article by Rocha et al. on a long-term, prospective cohort study of the neuropsychiatric outcomes of 65 intensive care unit (ICU) survivors assessed using the Mini Mental State Examination (MMSE), Hospital Anxiety and Depression Scale (HADS), Impact of Event Scale-6 (IES-6), and several wet inflammatory biomarkers.^(1)^ *Delirium* and elevated interleukin (IL)-10 and IL-6 were found to predict long-term cognitive impairment, IL-6 was associated with depression, and mechanical ventilation, IL-33, and C-reactive protein were associated with anxiety.^(1)^ It was concluded that cognitive impairment, depression, anxiety, and posttraumatic stress disorder may be complications of the ICU stay and that these outcomes may be associated with inflammatory markers.^(1)^ The study is impressive, but some points require discussion.

The first limitation of the study is that no cerebral imaging results were presented. Knowledge of cerebral imaging results is important for assessing the morphological background of neuropsychiatric dysfunction in ICU survivors. In ventilated and sedated patients, cerebral diseases that emerge during the ICU stay can easily be missed if imaging is not performed. Especially when they manifest only with cognitive impairment or mood disorders, cerebral lesions due to stroke, bleeding, inflammation, or hypoxia can be easily missed. It would be helpful to correlate the results of the MMSE, HADS, IES-6, and wet inflammatory biomarkers with imaging findings.

A second limitation is that the current medication and the medications that the included patients received during the ICU stay were not included in the analysis. Several drugs, particularly those with neurotropic effects, are known to cause neuropsychiatric dysfunction. These include sedatives, antidepressants, neuroleptics, pain killers, antiseizure drugs, and antibiotics.

A third limitation is that electroencephalograms were not recorded. Seizures can only manifest with neurocognitive impairment.^(2)^ In addition, *delirium* can be a clinical manifestation of seizure activity. Therefore, it is imperative to record electroencephalograms for all patients to avoid missing non-motor seizure activity.

A fourth limitation is that neuropsychological status was assessed only with the MMSE.^(1)^ To accurately assess which cognitive areas are impaired, a detailed, comprehensive neuropsychological examination is essential. According to the American Association of Psychiatry, six cognitive domains should be assessed: memory and learning, language, executive function, complex attention, social cognition, and perceptual and motor function.^(3)^ These tests are necessary to differentiate cognitive impairment and depression.

One inclusion criterion was patient consent.^(1)^ How was it possible to obtain consent for the study from people with *delirium* or cognitive dysfunction? Who agreed to the study in these cases? Who judged whether the patients had legal capacity?

Overall, this interesting study has limitations that call into question the results and their interpretation. Clarifying these weaknesses would strengthen the conclusions and add value to the study. The assessment of neuropsychiatric dysfunction after an ICU stay requires clinical neurologic examination, cerebral imaging, electroencephalogram recordings, and detailed neuropsychological testing.

## References

[1] Rocha FR, Gonçalves RC, Prestes GS, Damásio D, Goulart AI, Vieira AA (2023). Biomarkers of neuropsychiatric dysfunction in intensive care unit survivors: a prospective cohort study. Crit Care Sci.

[2] Kaplan PW (2003). Delirium and epilepsy. Dialogues Clin Neurosci.

[3] American Academy of Clinical Neuropsychology (2007). American Academy of Clinical Neuropsychology (AACN) practice guidelines for neuropsychological assessment and consultation. Clin Neuropsychol.

