# Supplementary material for: To: Biomarkers of neuropsychiatric dysfunction in intensive care unit survivors: a prospective cohort study
Source: Crit Care Sci. 2024 Jun 5;36:e20240260en. doi: 10.62675/2965-2774.20240260-en (PMC11152440; doi:10.62675/2965-2774.20240260-en)
Supplement: Translation

# Translation: Para: Biomarcadores de disfunção neuropsiquiátrica em sobreviventes de unidades de terapia intensiva: um estudo de coorte prospectivo

**DOI:** 10.62675/2965-2774.20240260-pt

**Authors:** Josef Finsterer, Fulvio Alexandre Scorza

**Affiliations:** 1 Viena Áustria Neurology and Neurophysiology Center - Viena, Áustria.; 2 São Paulo SP Brasil Disciplina de Neurociência, Escola Paulista de Medicina, Universidade Federal de São Paulo - São Paulo (SP), Brasil.

**Ao Editor**

Lemos com interesse o artigo de Rocha et al., um estudo de coorte prospectivo sobre os desfechos neuropsiquiátricos de 65 sobreviventes de unidades de terapia intensiva (UTI), avaliados pelo Miniexame do Estado Mental (MEEM), pela Escala Hospitalar de Ansiedade e Depressão (HADS - *Hospital Anxiety and Depression Scale*), pela Escala de Impacto do Evento-6 (IES-6 - *Impact of Event Scale-6*) e por vários biomarcadores inflamatórios solúveis.^(1)^ *Delirium* e concentrações elevadas de interleucina (IL)-10 e 6 foram considerados preditores de comprometimento cognitivo de longo prazo; a IL-6 foi associada à depressão; e ventilação mecânica, IL-33 e proteína C-reativa foram associadas à ansiedade.^(1)^ Foi concluído que comprometimento cognitivo, depressão, ansiedade e transtorno de estresse pós-traumático podem ser complicações de internação na UTI e que esses desfechos podem estar associados a marcadores inflamatórios.^(1)^ O estudo é surpreendente, mas alguns pontos precisam ser discutidos.

A primeira limitação do estudo é que não foram apresentados resultados de exames de imagem cerebral. O conhecimento desses resultados é importante para avaliar o contexto morfológico de disfunção neuropsiquiátrica em sobreviventes de UTI. Em pacientes ventilados e sedados, doenças cerebrais que aparecem durante a internação na UTI podem facilmente passar despercebidas se exames de imagem não forem realizados. Especialmente quando se manifestam apenas por meio de comprometimento cognitivo ou transtornos de humor, lesões cerebrais causadas por acidente vascular cerebral, sangramento, inflamação ou hipóxia podem facilmente passar despercebidas. Seria útil correlacionar os resultados do MEEM, da HADS, da IES-6 e dos biomarcadores inflamatórios solúveis com os achados de imagem.

A segunda limitação é que os medicamentos em uso e os que os pacientes receberam durante a internação na UTI não foram inseridos na análise. Diversos medicamentos, especialmente aqueles com efeitos neurotrópicos, são conhecidos por causar disfunção neuropsiquiátrica. Entre eles estão sedativos, antidepressivos, neurolépticos, analgésicos, anticonvulsivantes e antibióticos.

A terceira limitação é que os eletroencefalogramas não foram registrados. As convulsões só podem se manifestar com comprometimento neurocognitivo.^(2)^ Além disso, o *delirium* pode ser uma manifestação clínica de atividade convulsiva. Portanto, é imprescindível registrar eletroencefalogramas de todos os pacientes, para evitar que atividades convulsivas não motoras passem despercebidas.

A quarta limitação é que o estado neuropsiquiátrico foi avaliado somente com o MEEM.^(1)^ Para avaliar com precisão quais áreas cognitivas estão prejudicadas, é essencial que seja feita uma avaliação neuropsicológica detalhada e compreensível. De acordo com a *American Association of Psychiatry*, seis domínios cognitivos devem ser analisados: memória e aprendizado, linguagem, função executiva, atenção complexa, cognição social e função perceptomotora.^(3)^ Esses testes são necessários para diferenciar comprometimento cognitivo de depressão.

Um critério de inclusão foi o consentimento dos pacientes.^(1)^ Como foi possível obter o consentimento de pessoas com *delirium* e disfunção cognitiva? Quem autorizou a participação nesses casos? Quem julgou se os pacientes tinham capacidade legal?

No geral, o estudo é interessante, mas apresenta limitações que colocam em questão os resultados e suas interpretações. Questionar essas limitações reforçaria as conclusões e melhoraria a qualidade do estudo. A avaliação de disfunção neuropsiquiátrica depois de uma internação na UTI requer avaliação neurológica clínica, exames de imagem cerebral, registros de eletroencefalograma e avaliações neuropsicológicas detalhadas.
